# Sensor-Based Nerve Compression Measurement: A Scoping Review of Current Concepts and a Preclinical Evaluation of Commercial Microsensors

**DOI:** 10.3389/fbioe.2022.868396

**Published:** 2022-07-11

**Authors:** Simeon C. Daeschler, Rebecca Wienbruch, Catalina Bursacovschi, Kim Sophie Zimmermann, Selam Bekure Nemariam, Leila Harhaus, Ulrich Kneser, Alfons Dehé, Achim Bittner

**Affiliations:** ^1^ Department of Hand, Plastic and Reconstructive Surgery, Burn Center, University of Heidelberg, BG Trauma Hospital, Ludwigshafen, Germany; ^2^ Department of Plastic and Hand Surgery, University of Heidelberg, BG Trauma Hospital, Ludwigshafen, Germany; ^3^ SickKids Research Institute, Neuroscience and Mental Health Program, Toronto, ON, Canada; ^4^ Hahn-Schickard-Society for Applied Research, Villingen-Schwenningen, Germany; ^5^ University of Freiburg, Freiburg, Germany

**Keywords:** nerve compression, compression neuropathy, nerve entrapment, pressure measurement, pressure sensor, diabetic neuropathy

## Abstract

**Background:** Chronic nerve compression is the most common indication for nerve surgery. However, the clinical diagnosis still relies on surrogate parameters since devices for direct nerve compression pressure measurement (DNCPM) are clinically unavailable yet.

**Objectives:** To review previous approaches to DNCPM and evaluate presently available microsensor systems for their feasibility and reliability in preclinical nerve compression models.

**Methods:** A scoping literature review was conducted in accordance with the PRISMA-ScR guidelines. A subsequent market research aimed at identifying commercially available sensor systems potentially suitable for DNCPM. Sensors were evaluated for feasibility and safety of perineural sensor positioning, tissue compatibility and measurement reliability in a synthetic nerve compression model and an *ex-vivo* chicken leg model.

**Results:** A scoping literature review identified 197 potentially eligible studies of which 65 were included in the analysis. Previous approaches to DNCPM predominantly used pressure sensing catheters designed for fluid- or intra-compartmental pressure measurement. A market research identified two piezoresistive sensor systems (IntraSense, SMi, United States; Mikro-Cath, Millar, United States) as potentially suitable for intraoperative DNCPM. In both preclinical models, the detected compression pressure differed significantly between sensors and systems showed substantial measurement variability with a median percent coefficient of variation between 15.5% and 32%. Sensor position was accountable for up to 99.1% of the variance.

**Conclusion:** Measurement variability caused by unreliable sensor positioning is a key limitation of presently available sensors when applied for nerve compression measurements. Redesigned systems with small, flat-shaped and longitudinally oriented sensors and dedicated introducers would facilitate sensor positioning and therefore may allow for reliable measurements.

## Introduction

Peripheral nerves are susceptible to compression(Rydevik et al., 1981; Dahlin et al., 1984). Chronic nerve compression by neighboring anatomic structures may lead to segmental demyelination, axonal degeneration and intraneural fibrosis resulting in progressive numbness, tingling and weakness, collectively referred to as compression neuropathy(Dawson, 1993). The most common nerve entrapment sites are the carpal tunnel with an incidence of around 370 per 100,000 person years(Gelfman et al., 2009), and the cubital tunnel with an incidence of 30 per 100,000 person years respectively(Osei et al., 2017). In the United States alone, over 500,000 carpal tunnel release surgeries per year accumulate to an economic burden of more than 2.5 billion annually (Fajardo et al., 2012; Hubbard et al., 2018).

Despite being the most common cause for neuropathy, to date, nerve compression cannot be confirmed or quantified by direct nerve compression pressure measurement (DNCPM) because suitable pressure sensing probes are clinically unavailable. Therefore, nerve compression is commonly diagnosed via indirect clinical indications for focal nerve dysfunction. This may include a reduced signal conduction capacity through the entrapment site(Kane and Oware, 2012) and clinical tests for provoking typical symptoms including tingling, pain, or numbness in the skin area supplied by the nerve(Ho and Braza, 2021). These tests allow for approximate localization of the entrapment site but usually cannot reveal the specific anatomic structure that causes the entrapment. High-resolution ultrasonography can provide useful cues for identifying nerve entrapment by detecting nerve swelling proximal to the entrapment site(Klauser et al., 2009). However, critical thresholds differ significantly among studies(Koenig et al., 2009) and longitudinal distribution of nerve edema may similarly render exact localization challenging particularly in the context of multiple compression sites. Therefore, for numerous compression neuropathies, including the pronator syndrome(Presciutti and Rodner, 2011) and cubital tunnel syndrome(Palmer and Hughes, 2010), it is still a matter of debate which structure(s) may cause the entrapment and require surgical release. The relative proximity of potential nerve compression sites in the upper extremity, such as the medial intermuscular septum, the anconeus muscle, the cubital tunnel, and proximal arch of the flexor carpi ulnaris for the ulnar nerve, and the ligament of Struther, the bicipital aponeurosis, the pronator teres, and the fibrous arch of the supinator for the median nerve may further complicate the exact diagnosis. In clinical routine, this uncertainty may lead to unnecessarily extensive or insufficient surgical release in some patients and therefore may increase the risk of complications beyond what would be necessary for an effective treatment.

As a complementary diagnostic tool, DNCPM could provide definitive evidence for focal nerve entrapment with high spatial resolution. Suitable pressure sensing probes could be introduced in the presumed entrapment site to confirm the diagnosis prior to surgical release and reliably identify the compressing structure(s). This could be done intraoperatively under visual control or ultrasound guidance. For clinical research, the precise quantification of the compression pressure could serve as a yet unavailable gold standard reference for validating less invasive diagnostic procedures such as ultrasound and magnetic resonance imaging and therefore may help to reliably confirm nerve entrapments in neuropathic patients.

Here we conducted a scoping review of the literature to identify previous approaches and potential challenges associated with DNCPM. The results of this review informed a subsequent market research for commercially available suitable sensor systems, and two potentially suitable systems were selected and evaluated for feasibility and reliability in preclinical nerve compression models aiming to identify their current limitations and propose possible solutions.

## Materials and Methods

### Scoping Literature Review

A scoping literature review was conducted to map current key concepts for sensor-based perineural pressure measurement, define sensor requirements and identify potential gaps in research. Based on the PRISMA-ScR guidelines(Tricco et al., 2018)**,** a systematic search strategy was designed in December 2020 for the PubMed database to identify all potentially relevant studies. The date of the last search was 26 January 2021. The search results were screened against a predetermined checklist of inclusion criteria (Supplementary Table S1) and studies not meeting these criteria were excluded. Further, the reference list of included studies was screened for potentially eligible publications. Included were all studies that reported sensor-based, direct perineural pressure measurements.

### Scoping Market Research

Based on the results of the review, a scoping market research was conducted to identify potentially suitable micro sensor systems for DNCPM.

### Preclinical Evaluation of Commercial Sensors

#### Sensor Selection and Set up

Based on the results of the review and the market research, two piezoresistive sensors for medical applications, the Mikro-Cath (Millar, Houston, TX, United States) and the IntraSense (SMi, Milpitas, CA, United States) were selected for further preclinical testing.

The IntraSense was evaluated with a SMi evaluation kit (Silicon Microstructures Inc., Milpitas, CA, United States) including an Arduino microcontroller (Arduino SA, Lugano, Switzerland) and an I2C module. The Arduino readout code was optimized for the purpose of the experiments and implemented in MATLAB (Mathworks, Natick, MA, United States) for automated measurements.

For the Mikro-Cath, an Arduino Uno board (Arduino SA, Lugano, Switzerland) with an instrumentation amplifier (AD620 instrumentation amplifier, Analog Devices, Wilmington, MA, United States) and the BME-680 module (Bosch Sensortec GmbH, Reutlingen, Germany) were used. The Mikro-Cath measurements were performed using the Arduino IDE 1.8.12 software (Arduino SA, Lugano, Switzerland) and subsequently analyzed in a custom-written MATLAB program using version R2020a with the Arduino Support Package (MathWorks, Natick, MA, United States).

#### Preclinical Nerve Compression Models

**FIGURE 1:**
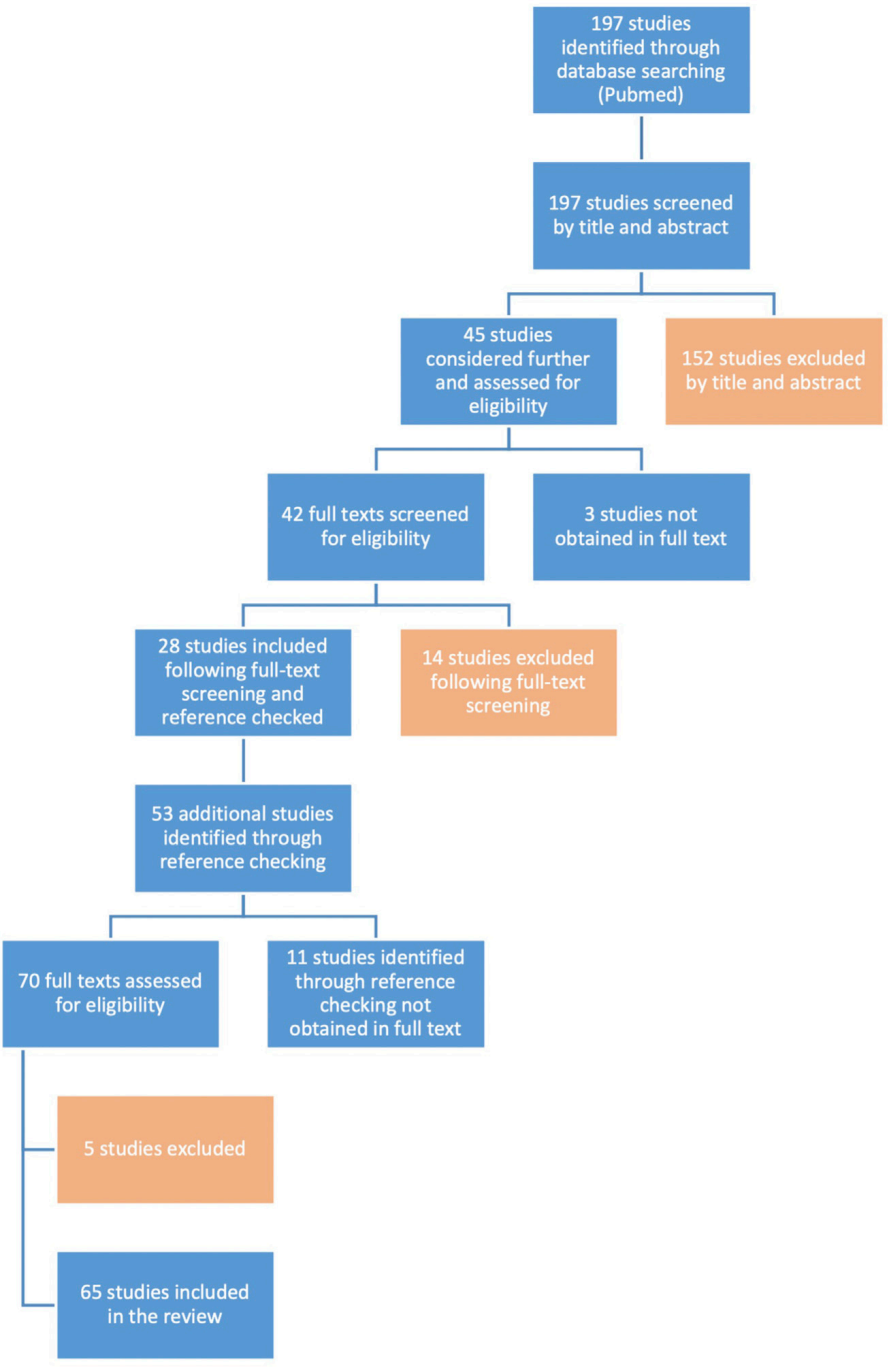
Flow diagram. Visualization of the literature search and the study selection process according to the PRISMA-ScR guidelines.

**FIGURE 2:**
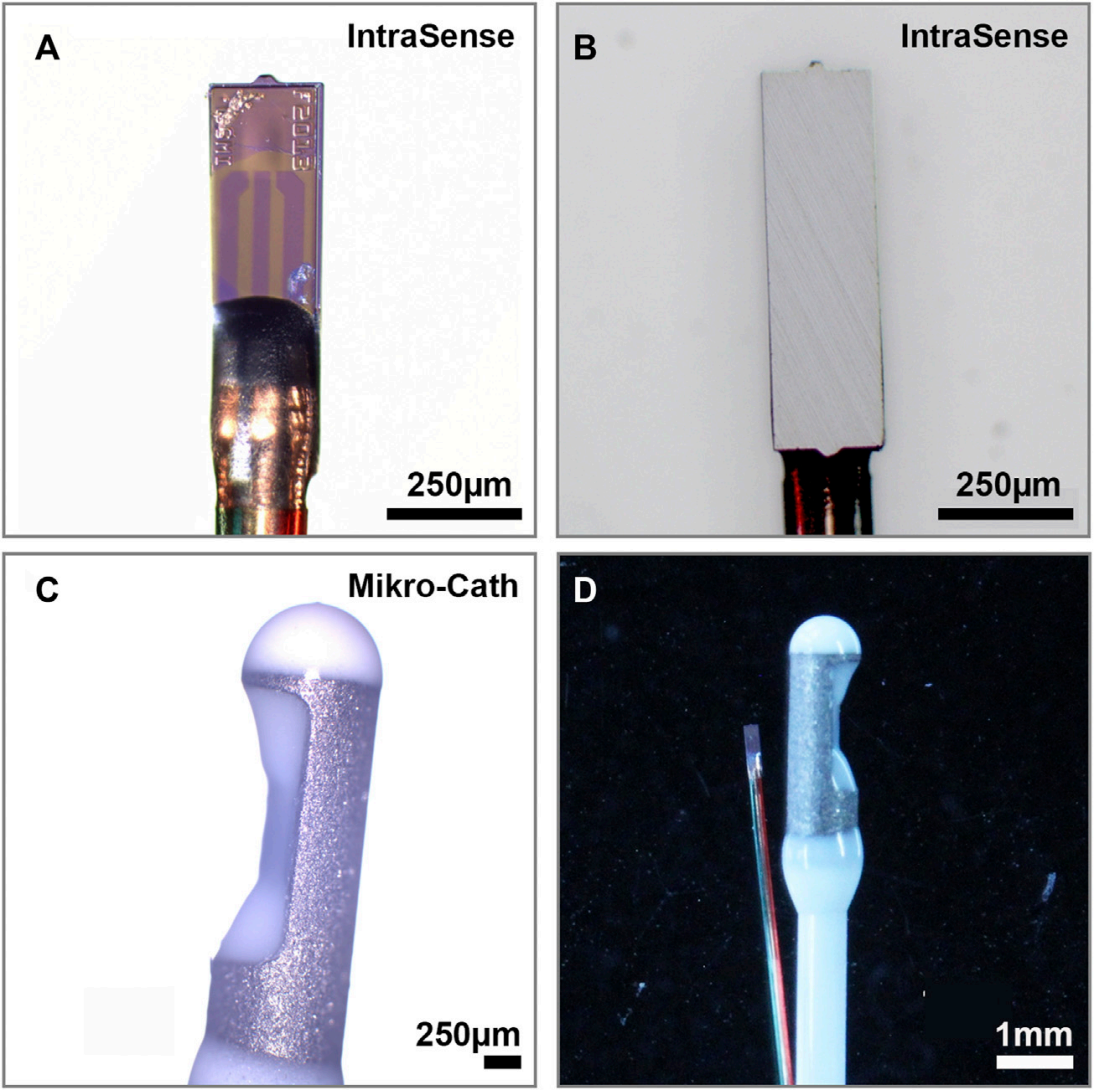
Pressure Sensors **(A)** SMi’s IntraSense is a disposable piezoresistive absolute pressure sensor with a pressure sensitive membrane close to the tip and a **(B)** metal undersurface (750 × 220 × 75 µm) that can be integrated into a 0.33 mm hypo tube **(C)** Millar’s Mikro-Cath is a CE certified, and FDA approved disposable piezoresistive relative pressure sensor with a pressure sensitive membrane integrated into an indentation just below the catheter tip **(D)** Side to side comparison reveals the size differences between both sensor elements.

We used two experimental models to evaluate feasibility of sensor positioning, reliability, and measurement variability for both sensors. To simulate a nerve entrapment site under consistent conditions we used a synthetic nerve (SynDaver, Tampa, United States) that was compressed by an elastic polyester strap (20 × 3 × 0.1 cm) exerting a constant pressure on the nerve (Figure 1A–C). The calibrated sensors were introduced and positioned between the nerve and the elastic strap for measuring the nerve compression pressure. In addition, a chicken leg *ex vivo* model was used to simulate close-to-reality tissue conditions for DNCPM. Using a muscle split technique at the proximal thigh, a minimally invasive access-path to the sciatic nerve was carefully dissected within anatomical planes. Then, the sensor was advanced along the nerve in distal direction to position the sensor tip between the epineurium and the adjacent fibro-muscular tissue (Figure 2A).

Once the sensor was positioned the pressure was continuously recoded for 1000 consecutive measurements over approximately 2 min. The average pressure for each position was calculated. Then, the sensor was fully removed, the experimenter was instructed to recreate the same sensor position and the measurement series was repeated for a total of ten times. The feasibility of perineural positioning was rated and the tissue compatibility of the sensor components were determined via light microscopy (Axio Imager. M2, Carl Zeiss Microscopy GmbH, Jena, Germany).

### Statistical Analysis

Statistical analyses were conducted using JMP (version 15.1.0; SAS Institute). All means are expressed with standard deviation (±SD). Non-normally distributed data is presented as median with 95% confidence intervals. To compare measurements between positions of the same sensor a Wilcoxon test was used. To quantify measurement variation for each sensor position, the percent coefficient of variation (%CV) as the percentage of the mean represented by the standard deviation was calculated. A variance component analysis was conducted to estimate the relative contribution of sensor position on measurement variability. An alpha level of 0.05 was used.

## Results

### Scoping Literature Review

#### Search Results

The systematic literature search identified a total of 197 potentially eligible studies on perineural pressure measurements (see Supplementary material for detailed search strategy). After title and abstract screening, 45 studies were full-text screened and an additional 53 studies were identified by reference checking. In total, 65 studies fulfilled the inclusion criteria following full-text screening (Supplementary Table S1). Figure 1 illustrates the study selection process.

#### Study Characteristics

The majority of the included studies measured the compression pressure on the median nerve in the carpal tunnel (*n* = 54; 83,1%), followed by the ulnar nerve (*n* = 5; 7,7%) in the cubital tunnel or in Guyon’s canal. Other studies included measurements on other upper extremity nerves, on cervical nerves or on the sciatic nerves. Most studies were performed clinically (n = 48; 73,8%), whereas others involved human cadavers (n = 16; 24,6%). One experimental study was conducted on rabbits(Diao et al., 2005). A detailed presentation of all included studies can be found in the Supplemental material.

#### Previous Approaches to Nerve Compression Measurement

The most frequently applied method for perineural DNCPM utilized fluid filled catheters(Tanzer, 1959; Gelberman et al., 1981; Werner et al., 1983; Goodman et al., 2001; Okutsu et al., 2004; Werner et al., 2004; Diao et al., 2005; Sanz et al., 2005; Fuller et al., 2006; Keir et al., 2007; Rempel et al., 2008) connected to an external pressure transducer, as pioneered by Tanzer et al., in 1959(Tanzer, 1959). Commonly employed catheters were angiocatheters(Hamanaka et al., 1995; Okutsu et al., 2004), angioplasty catheters(Diao et al., 2005), multi-perforated catheters(Keir et al., 2007; Rempel et al., 2008) or fluid filled systems developed for intracompartmental pressure monitoring(Luchetti et al., 1998; Werner et al., 2004). To measure the perineural pressure, the catheter was transcutaneously or intraoperatively introduced into the constriction site and immediately removed after the measurement. More recently, digital systems with tip mounted pressure sensors, initially developed for intracranial or intracompartmental pressure monitoring, were applied for DNCPM, in an effort to overcome drawbacks of fluid filled tubes and extracorporeal transducers (Schuind, 2002; Ikeda et al., 2006; Uchiyama et al., 2010).

#### Key Challenges and Sensor Requirements

Previously reported approaches almost exclusively diverted systems that have been developed for closed tissue compartments or fluid filled cavities. Using those systems for DNCPM assumes an equally distributed pressure profile within the constriction site and potentially neglects local pressure peaks on mechanically entrapped nerves. Capturing pressure peaks along the nerve, requires the sensor to be positioned in a plane between the constricting structure and the nerves’ surface. This generally requires sterile, biocompatible sensors with suitable geometric dimensions and sensitivity in the anticipated pressure range. The sensor must allow for safe introduction into the constriction site, often without visual control. Further, the sensor surface should be positioned perpendicular to the main force vector and ideally detect local pressure maxima over a 2–3 cm distance along the nerve. Easy-to-read depth and orientation markings would therefore facilitate standardized catheter placement. Presently commercially available sensor systems for clinical bedside application include fiber optic, capacitive, piezoelectric and piezoresistive sensors. However, to date, systems that were specifically developed for nerve compression measurements are commercially unavailable.

### Results of the Scoping Market Research

Based on size, shape, pressure range and availability, two commercial piezoresistive sensor systems were considered potentially suitable for nerve compression measurements and selected for preclinical evaluation. Two additional potentially suitable sensors (NovaSensor P330W, Amphenol, Conneticut, United States; and Ultra-miniature fiber optical pressure sensor, Samba Sensors AG, Goteborg, Sweden) have been excluded from further analysis because of lacking availability.

#### Sensor Characteristics

**FIGURE 3:**
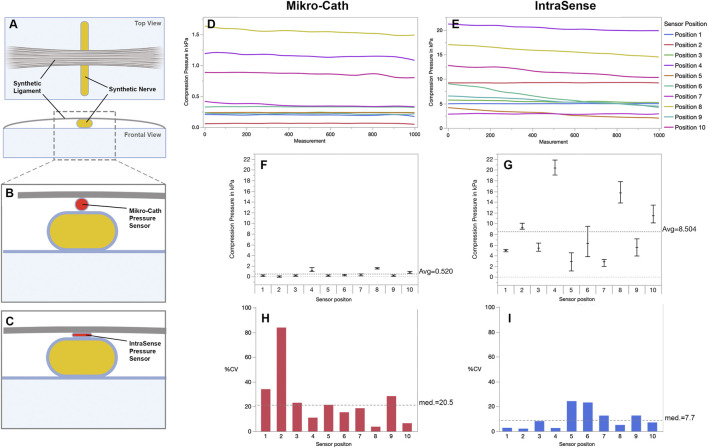
Perineural pressure measurement in a synthetic nerve compression model **(A)** Experimental set-up using a synthetic nerve and ligament to simulate nerve entrapment **(B,C)** The sensor is inserted into the entrapment site and positioned between nerve and ligament to measure the pressure that is exerted on the nerve **(D,E)** Serial nerve compression pressure measurements in ten individual sensor positions. Even though the examiner tried to recreate the same sensor position each time, the detected pressure differed significantly between positions **(F)** The average compression pressure (±SD) per sensor position measured with the Mikro-Cath and **(G)** Intrasense respectively. Notably, the flat shaped Intrasense measures consistently higher pressure values as compared to the Mikro-Cath **(H)** Measurement variability of the Mikro-Cath and **(I)** Intrasense expressed as precent coefficient of variation (%CV) for each individual sensor position, indicating greater variability in Mikro-Cath measurements. Figures were designed with Biorender.com.

SMi’s IntraSense is a disposable piezoresistive absolute pressure sensor. This device is presently not FDA approved and not integrated into a catheter. With a size of 750 × 220 × 75 µm and a rectangular, flat shape (Figure 3A), it can be integrated into a 1-french (F) hypo tube (diameter 0.33 mm). The pressure sensitive membrane is located on the flat surface of the sensing element (Figure 3B). The operating pressure ranges from -40–67 kPa and the operating temperature ranges from 10 to 70°C with a supply voltage of 3.3 or 5 V (volts). The sensing element is connected to an I2C module via insulated copper wires. The sensor is operated by SMi’s evaluation kit consisting of an Arduino microcontroller and an Arduino-based measurement program.

Millar’s Mikro-Cath is a disposable piezoresistive relative pressure sensor. This device is CE certified and FDA approved(Inc, 2018) for cardiovascular, intra-compartmental and airway pressure measurements and integrated into a 3.5F catheter (1.2 mm) with a circular cross section and rounded tip. The pressure sensitive membrane is integrated into an indentation at the side of the catheter (Figures 2C,D). The operating pressure ranges from 6.7 to 40 kPa and the operating temperature ranges from 15 to 40 °C with a supply voltage of 5.0 V (volts). To calibrate the Mikro-Cath, the ambient air pressure must be measured prior to each set of measurements with a separate device. The Mikro-Cath is compatible with standard patient monitoring systems via interface cables and a Millar pressure control unit.

### Preclinical Evaluation of Commercial Pressure Sensors

#### Sensor Biocompatibility

Intraoperative DNCPM require the sensor surface to be sterile, stable, and non-toxic to the surrounding tissue. The Mikro-Cath comes as a sterile single use device and is CE and FDA approved for short term limited body contact below 24 h (Inc, 2018). The IntraSense is presently classified as an experimental device and its sensor surface has not yet been approved for sterilization or clinical use. To determine tissue compatibility we microscopically assessed the surface of both sensors for signs of mechanical abrasion, damage, or surface reaction after prolonged muscle and nerve tissue contact for approximately 30min in a chicken leg model. The IntraSense showed signs of tissue apposition and surface abrasion, indicating poor biocompatibility, risk of toxicity and tissue damage induced by the sharp sensor edges such as inadvertent nerve trauma during sensor positioning. In contrast, the Mikro-Cath had no visible abrasion, damage, or apposition after prolonged tissue exposure.

#### Feasibility of Perineural Sensor Positioning

To quantify the pressure that surrounding tissues exerts on peripheral nerves in anatomic constriction sites, the sensor must be advanced along the nerve and positioned between the nerve and the compressing structure. The sensor size, form, and the stiffness and caliber of the attached catheter or introducer determine the feasibility of the sensor positioning and the risk of inadvertent tissue damage. The Mikro-Cath provided sufficient stiffness to advance the sensor within anatomical planes and features a rounded tip that may reduce the risk of tissue damage. However, due to its round cross section, the sensor was easily displaced from the compression site towards the side of the nerve. Further, rolling of the catheter along its axis resulted in an oblique force vector on the sensor surface, potentially affecting the detected pressure. In contrast, the IntraSense provides a flat sensor shape that facilitated sensor positioning and maintained perpendicular force vectors. However, its angular tip shape and the flexible catheter complicated sensor advancement and precise positioning.

#### Reproducibility and Variability of Nerve Compression Measurements

To determine the reproducibility of perineural DNCPM for both sensors, we used an experimental nerve compression model that provided a consistent compression pressure on a synthetic nerve (Figures 3A–C). The sensors were carefully positioned within the constriction site and 1000 consecutive measurements were recorded over approximately 2 min for a total of ten sensor positions.

The larger Mikro-Cath sensor measured an average compression pressure of 0.52 ± 0.47 kPa among all ten sensor positions. The mean compression pressure differed significantly among sensor positions (Wilcoxon test *p* < 0.001), ranging by factor 26 from 0.06 ± 0.05 kPa to 1.54 ± 0.06 kPa (Figures 3D,F). Variance components analysis showed that sensor position was accountable for 98% of the measurement variation (*p* < 0.0001).

In the same model, the smaller, flat shaped IntraSense sensor detected an overall average compression pressure of 8.5 ± 5.53 kPa (Figures 3E,G) ranging by factor 7 among sensor positions (2.9 ± 0.36 kPa to 20.4 ± 0.54 kPa; Wilcoxon test *p* < 0.001) and sensor position being accountable for 98.5% of the variation (*p* < 0.0001).

To quantify the variability of serial measurements over time while the sensors were kept in a constant position, we calculated the percent coefficient of variation (%CV) as the percentage of the mean represented by the standard deviation for each position and sensor. For the Mikro-Cath the median %CV among all ten sensor positions was 20.5%, indicating that the standard deviation equaled 20.5% of the respective average compression pressure (95% CI 8.5%–41.9%; Figure 2H). For IntraSense measurements, the median %CV was 7.69% (95% CI 4.3%–15.3%: Figure 2I).

**FIGURE 4:**
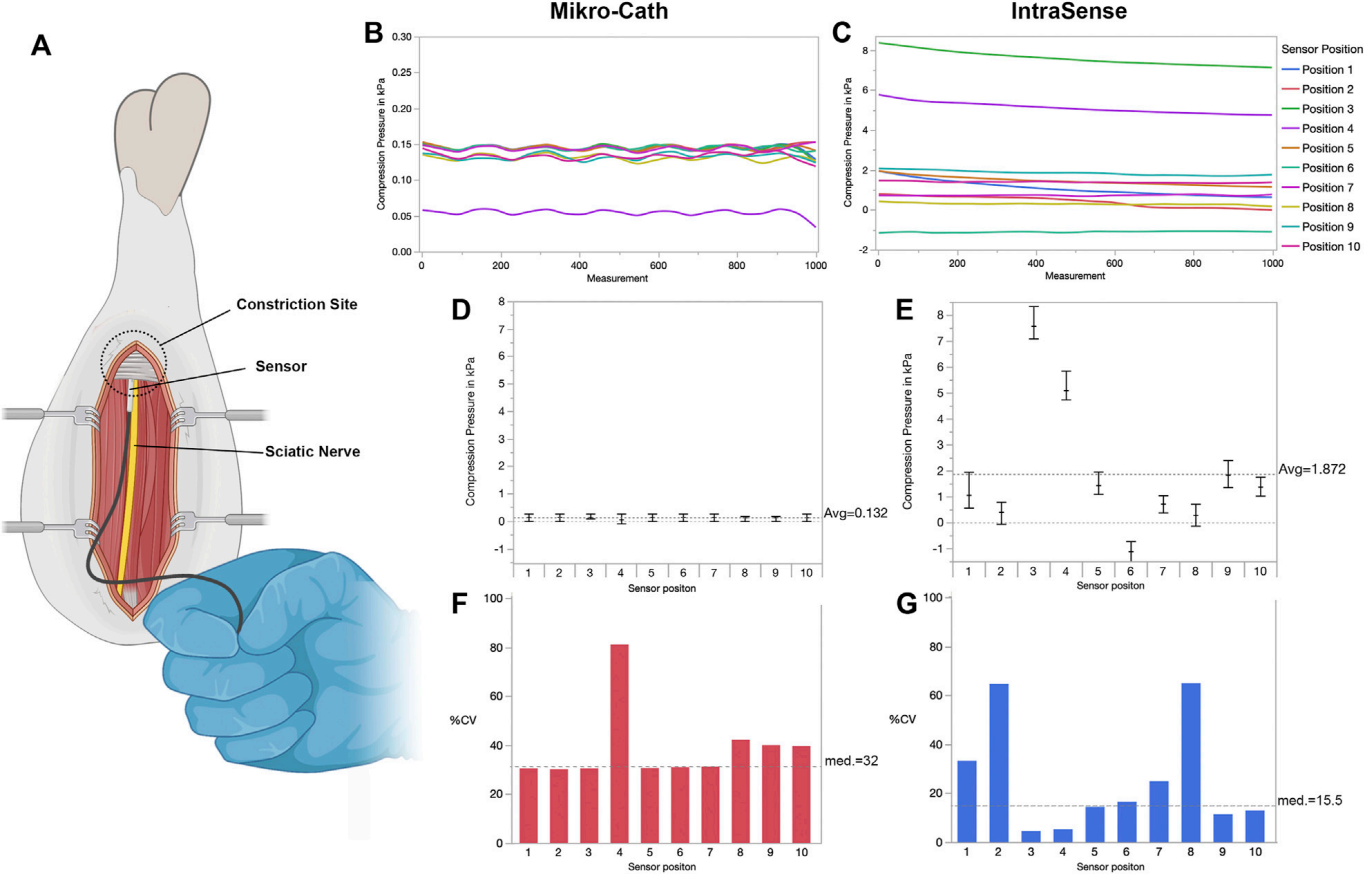
Perineural pressure measurement in an *ex vivo* chicken leg model **(A)** Experimental set-up with the surgically exposed sciatic nerve in the chicken thigh. The sensor is advanced along the nerve and positioned between the nerve and adjacent fibromuscular tissue **(B,C)** Within the constriction site, serial nerve compression pressure measurements were performed in ten positions for each sensor. The examiner was instructed to recreate the same sensor position each time. Similar to the synthetic nerve compression model, the recorded pressure differed significantly between positions **(D)** The average compression pressure and standard deviation per sensor position measured with the Mikro-Cath and **(E)** IntraSense respectively. Of note, accidental displacement or rotation of the senor may lead to negative pressure readings as shown in position 6 with the IntraSense device **(F)** Both sensors, the Mikro-Cath and **(G)** IntraSense show considerable measurement variability expressed as precent coefficient of variation (%CV) for each individual sensor position. Figures were designed with Biorender.com.

Although the synthetic nerve compression model provided a standardized set up with a consistent compression pressure, the extent to which it reflects real-world tissue conditions is limited. We therefore used an *ex vivo* chicken leg model to test both sensors in conditions with realistic biomechanic tissue characteristics (Figure 4A). In a similar procedure, a total of ten sensor positions were assessed with 1000 consecutive measurements each. The Mikro-Cath measured an overall average nerve compression pressure of 0.13 ± 0.06 kPa with the average pressure per sensor position ranging by factor 29 from 0.05 ± 0.04 kPa to 1.46 ± 0.05 kPa (Wilcoxon test *p* < 0.001; Figures 4B,D) and sensor position being accountable for 24.5% of the variation (*p* < 0.0001).

In the same chicken leg, the flat-shaped IntraSense measured an overall average compression pressure of 1.87 ± 2.44 kPa (Figure 4C). The position specific mean compression pressure ranged from -1.1 ± 0.18 kPa to 7.58 ± 0.34 kPa (Wilcoxon test *p* < 0.001; Figure 4E). Sensor position was accountable for 99.1% of the measurement variation (*p* < 0.0001). We then quantified the position specific measurement variability in the chicken leg model. The Mikro-Cath showed a median %CV of 32% (95%CI 28.3%–51.3%; Figure 4F) and the IntraSense 15.5% (95%CI 9.2%–41.4%; Figure 4G) among ten sensor positions respectively.

## Discussion

Compression neuropathies are common, yet sensor systems for measuring nerve compression pressure are clinically unavailable. Here we present a scoping review of previous experimental concepts for direct nerve compression pressure measurements (DNCPM), we identify key challenges and sensor requirements and evaluate commercially available sensor systems for their applicability in preclinical nerve compression models.

A scoping review of the literature revealed that first attempts to quantify the nerve compression pressure within entrapment sites date back to the 1950s(Tanzer, 1959) and started to gain traction in the 1990s with over 30 published clinical and experimental studies to date (Supplemental material). The applied sensor concepts evolved over time. Initially, fluid-filled catheters or needles connected to external pressure transducers were used. Although widely employed for various clinical applications, those systems may suffer from pressure dynamics induced signal damping, inadvertent catheter occlusion or artifacts from pressure applied along the catheter. More recently, microsensor systems have been introduced for medical applications, now representing the standard for most FDA/EMA approved pressure monitoring devices. However, the presently available devices have been developed for other medical indications that necessitate pressure monitoring in enclosed, often fluid-filled spaces such as blood vessels, the subarachnoid space, the urinary tract, and others [15]. In these cavities the catheter probe is usually surrounded by a medium with a homogenous pressure profile exerting a constant pressure on each side of the sensor. Common nerve entrapment sites however, feature a distinct architecture and may rather be considered semi-enclosed spaces with local pressure maxima over the course of the mechanically entrapped nerve. Safely introducing a sensor element into these narrow entrapment sites to capture these local pressure maxima in a clinical setting therefore requires a different sensor design.

To date, the cost-effective manufacturing of piezoresistive microsensors enables their large-scale application in disposable, medical grade catheters. When applied for nerve compression measurements, however, our results indicate that presently available devices suffer from high measurement variability, mostly attributable to unreliable sensor positioning.

The CE certified and FDA approved(Inc, 2018) Mikro-Cath features a seamless integration into commonly used patient monitoring systems and therefore could be readily applied for intraoperative or bedside DNCPM. Further, compared to the flexible, angular IntraSense, the Mikro-Cath provided a sufficient catheter stiffness and a round tip to facilitate introduction of the sensor element between nerve and ligament. However, the Mikro-Cath is optimized for measurements in enclosed compartments, rendering its design less ideal to for application between solid structures. The recessed position of the sensor element within the lumen of the catheter impedes pressure transmission from stiff adjacent structures. Further, its tube-like shape causes the catheter to easily displace or rotate. This is in agreement with the results of our experiments. The Mikro-Cath detected consistently lower pressure values as compared to the IntraSense throughout the experiments and in both experimental models. despite the expected volume effect due to its 3.5-fold larger diameter. In addition, although the examiner aimed at consistently recreating the correct Mikro-Cath sensor position each time, the detected pressure ranged by factor 26 and 29 among positions in both applied nerve entrapment models respectively. Given the ideal experimental conditions with sensor positioning under visual control, these results highlight the limitations of currently available monitoring systems when applied for nerve compression pressure measurements.

The IntraSense presently represents a commercially available experimental device, without clincial approval, nonetheless offering several specifications that make this sensor potentially suitable for nerve compression measurement. The flat shaped, tip-mounted sensor surface facilitates sensor positioning in the correct tissue plane, and its small size reduces volume effects on pressure measurements or excessive strain on the adjacent nerve. Given the exposed sensor surface, the IntraSense detected consistently higher pressure values under the same conditions as compared to the larger Mikro-Cath (Figures 4B–E). However, despite its favorable shape and size, inadvertent dislocation still occurred, occasionally producing negative pressure readings (Figure 4E, sensor position 6). Accordingly, the IntraSense measurements showed considerable test-retest variability similar to the Mikro-Cath. In conjunction with the lacking biocompatibility of the sensor surface, this renders the IntraSense ineligible for clinical DNCPM in its currently available set-up.

Interestingly, in the *ex vivo* model, the Mikro-Cath pressure readings undulated around a constant mean (Figure 4B). Raw data analysis revealed that the detected pressure tended to jump between two consistent pressure values, indicating limited resolution of the sensor system in these very low pressure ranges (below 0.2 kPa). In contrast, the IntraSense measurements occasionally followed a negative slope over time (Figure 4C; positions 3, four and 5). This may be an artifact introduced by a slow, steady sensor movement, progressively changing the force vector on the sensor surface. This hypothesis is in agreement with the results of the variance component analysis, indicating that sensor position was accountable for up to 98 and 99% of the measurement variability for both tested sensors, respectively. Improving sensor positioning therefore represents the key to reliable DNCPM.

Aiming to replicate nerve entrapment in an experimental setting under controlled conditions, the chosen models feature several limitations. The synthetic nerve model offers consistent nerve compression pressure, and close-to-reality dimensions of large extremity nerves but lacks realistic tissue characteristics. The *ex vivo* chicken leg model on the other hand features realistic tissue conditions, allowing proper surgical dissection and the assessment of subsequent intraoperative sensor positioning. Nevertheless, no actual nerve entrapment was present in this model and the experiments were thus conducted under physiological pressure conditions. The lower pressure ranges may limit the translatability of the results, however the robust effect of sensor design and sensor position on pressure readings likely holds true for clinical application in nerve entrapment sites. This reinforces the need for redesigned sensors, specifically developed and assayed for detecting local pressure peaks along peripheral nerves.

Based on the results of the literature review and the conducted experiments, we propose a piezoresistive microsensor longitudinally mounted on the tip of a small, flat-shaped probe with a biocompatible and sterilizable packaging and easy-to-read depth and orientation markings on the sensor surface. Piezoresistive sensors are based on electrical resistivity which changes in response to static and dynamic pressure application on the sensor element. A thin, but sturdy wire-catheter can separate the probe from the signal processing electronics, and thus may allow for a miniaturized probe design and facilitate the precise positioning of the sensor element between adjacent structures in semi-enclosed nerve entrapment sites. Given the relatively minor changes compared to currently used sensors, the redesigned sensors can be cost-effectively manufactured in small dimensions.

A suchlike sensor may facilitate the clinical diagnosis in challenging cases of suspected nerve entrapment. Beyond that, the clinical value of DNCPM will be defined by their ability to contribute to our understanding of the pathophysiology of compression neuropathy and its potential role in chronic neuropathic disorders. Further, using suchlike sensitive sensors to precisely localize and quantify mechanical strain on peripheral nerves may inform surgical techniques for targeted nerve decompression. Arguably more important could be the role of DNCPM as a yet unavailable gold standard diagnostic tool that enables validation of non-invasive diagnostics (i.e., imaging techniques) and thereby, over time, ideally renders itself superfluous in clinical routine.

In conclusion, as specialized sensors for direct nerve compression measurements are commercially unavailable, other medical or experimental pressure sensing devices have been diverted in attempts to quantify nerve compression. However, we have demonstrated that these devices suffer from considerable measurement variability mainly attributable to their design and the consequently challenging sensor positing. Redesigned sensors, specifically developed for application in nerve entrapment sites may help to overcome these shortcomings and enable reliable nerve compression measurements in research and clinical routine.

## Data Availability

The raw data supporting the conclusion of this article will be made available by the authors, without undue reservation.

## Abbreviations

DNCPM, Direct nerve compression pressure measurement; CE, Conformité Européenne; FDA, United States Food and Drug Administration; EMA, European Medicines Agency.
