# Supplementary material for: Sensor-Based Nerve Compression Measurement: A Scoping Review of Current Concepts and a Preclinical Evaluation of Commercial Microsensors
Source: Front Bioeng Biotechnol. 2022 Jul 11;10:868396. doi: 10.3389/fbioe.2022.868396 (PMC9309797; doi:10.3389/fbioe.2022.868396)
Supplement: Supplementary file 1 [file Table1.docx]

|  | Inclusion criteria |
| --- | --- |
| Population | Peripheral nerves:   - healthy or affected by a type of neuropathy - from humans or animals - from living subjects or from cadavers - targeted specimens or whole bodies |
| Intervention | Measurement of perineural pressure using:   - FDA or EMA approved sensor systems - experimental sensor systems |
| Comparison | - not applicable |
| Outcome | - not applicable |
| Study design | - experimental *in vivo* study, human cadaver study or clinical trial - published in English or German |

**Supplementary Table 1.** Inclusion criteria for studies for this review. Criteria for inclusion are based on the PICOS model (population, intervention, comparison, outcome, study design) as recommended by the PRISMA-ScR guidelines(Tricco et al., 2018)
